# Amoebic encephalitis: case report and literature review of neuroimaging findings

**DOI:** 10.1259/bjrcr.20150499

**Published:** 2016-07-28

**Authors:** Matthew William Lukies, Yoshiyuki Watanabe, Tetsuo Maeda, Shinsuke Kusakabe, Hideyuki Arita, Noriyuki Tomiyama

**Affiliations:** ^1^Department of Diagnostic and Interventional Radiology, Osaka University Graduate School of Medicine, Suita, Japan; ^2^Department of Haematology and Oncology, Osaka University Graduate School of Medicine, Suita, Japan; ^3^Department of Neurosurgery, Osaka University Graduate School of Medicine, Suita, Japan

## Abstract

We present a fatal case of amoebic encephalitis due to *Acanthamoeba* spp. in an immunosuppressed male. Amoebic encephalitis can be a diagnostic challenge as clinical features are non-specific and imaging findings resemble other more common diagnoses such as tumours, haemorrhage or encephalitis from other causes. Here, we present the diagnostic imaging findings in this case and review the reported imaging findings in other cases throughout the literature.

## Clinical presentation

52-year-old immunosuppressed male presented with fever, impaired consciousness and headache. Past medical history comprised curatively resected gastric cancer (T1N0M0) complicated by post-operative pancytopenia and diagnosis of aplastic anaemia on bone marrow biopsy. Subsequent bone marrow transplantation led to graft versus host disease and, at the time of presentation, the patient had pancytopenia and was being treated with prednisolone.

## Investigations/imaging findings

**Figure 1.**
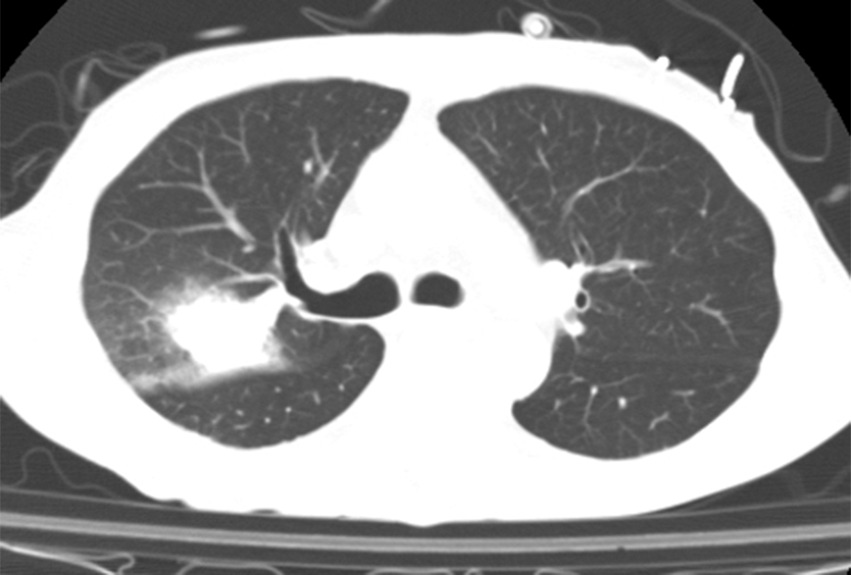
Axial chest CT image demonstrating a dense mass consolidation with surrounding ground-glass opacity in the right upper lobe.

**Figure 2.**
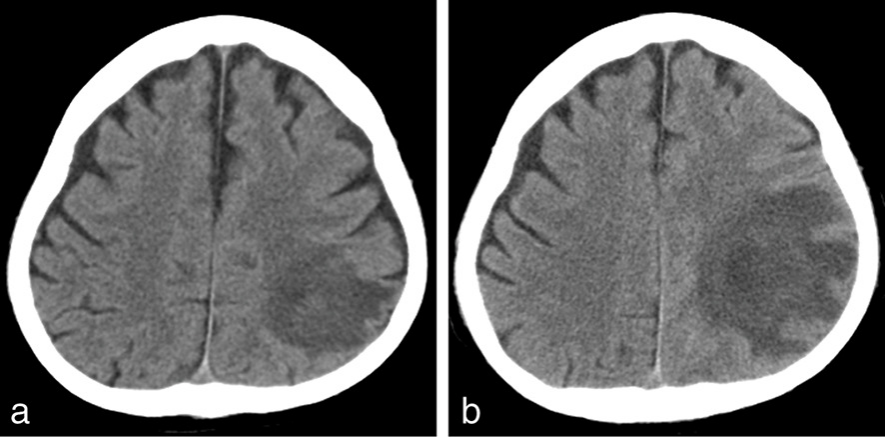
(a) Brain CT image demonstrating a low density mass in the left parietal lobe. (b) Brain CT image taken 2 days later demonstrating rapid growth of the low density mass.

CT scan of the chest (Figure 1) revealed a dense mass consolidation with surrounding ground-glass opacity in the right upper lobe. *Staphylococcus* was identified from blood cultures and *Stenotrophomonas maltophilia* from bronchiolar lavage, but pathological examination of the cerebrospinal fluid was unremarkable. Serial CT brain investigations (Figure 2) performed 2 days apart demonstrated a rapidly growing low density mass in the left parietal lobe.

**Figure 3.**
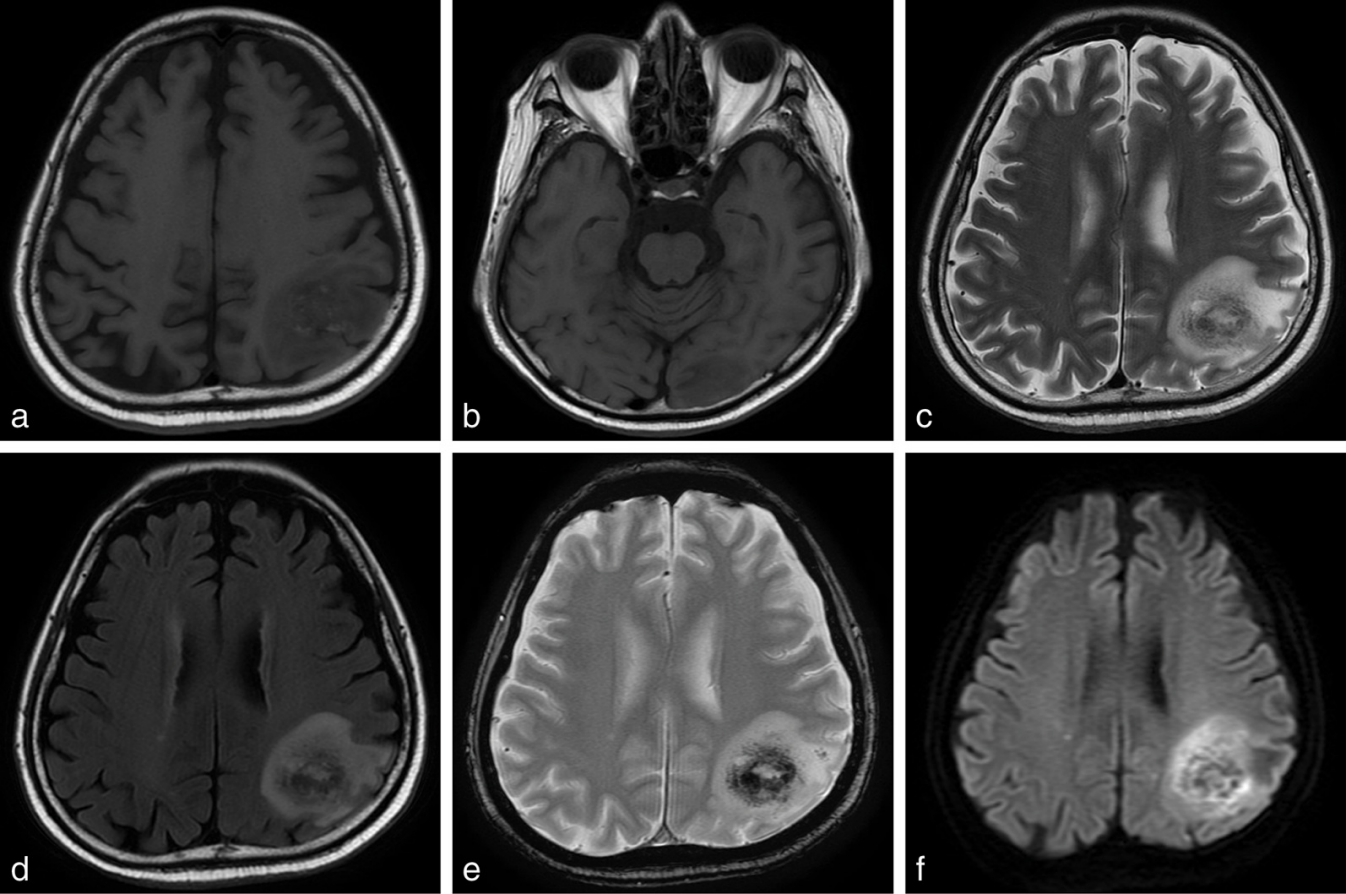
MRI of brain *T*_1_ (a, b), *T*_2_ (c), fluid-attenuated inversion recovery (d), *T*_2_ gradient echo (e) and diffusion-weighted images (f).

3.0 T MRI brain (Figure 3) was performed without contrast owing to gadolinium and iodine allergies. *T*_1_ weighted images revealed a large 50 mm pseudotumoral low signal dense area in the left parietal lobe with patchy central high signal, and a separate 33 mm low signal dense homogeneous area in the left occipital lobe. On *T*_2_ weighted images, the two masses were seen with a central area of high signal, low signal rim and surrounding oedema. *T*_2_ gradient echo images revealed stronger central low signal areas, indicating haemorrhagic change. Fluid-attenuated inversion-recovery (FLAIR) images demonstrated hyperintense lesions and there was restricted diffusion on diffusion-weighted imaging.

## Differential diagnoses

- Infective encephalitis—considered the primary differential.
- Tumour (primary or secondary from previous gastric cancer).
- Infarction—considered less likely as the cerebral cortex was preserved.
- Post-transplantation lymphoproliferative disease.
- Tumefactive demyelination.

## Treatment and outcome

The patient developed multiorganism septicaemia, with the pathogens identified including *Cryptococcus*, *Nocardia* and *Staphylococcal* species. A broad-spectrum antibiotic regimen was commenced comprising doripenem, linezolid, clindamycin and azithromycin, but the patient’s condition deteriorated further and he required intubation. Decompressive neurosurgery for cerebral oedema was carried out but unfortunately the patient died. Granulomatous amoebic encephalitis due to *Acanthamoeba* spp. was diagnosed on autopsy, with ameobic lesions also found in the lungs.

## Review imaging findings in case report literature

29 cases of amoebic encephalitis with written imaging findings in 18 publications were reviewed,^1–18^ including our current case. The demographics of the patients are shown in Table 1. Imaging findings across all case reports are summarized in Tables 2–4. Where a finding was not described in the case report, it was regarded as “not stated.”

**Table 1.** Demographics and causative organisms in 29 reviewed cases

Age (years)	Gender		Immune status		Organism	
0.5 – 75	Male	17 (59%)	Immunosuppression	11 (38%)	*Acanthamoeba* spp.	8 (28%)
28.7 (mean)	Female	12 (41%)	Chronic illness	4 (14%)	*Balamuthia mandrillaris*	17 (59%)
			Not clearly stated	3 (10%)	*Naegleria fowleri*	1 (3%)
			Previously well	11 (38%)	Ameoba species not clear	4 (14%)

**Table 2.** Summary of imaging findings in 29 reviewed cases

Lesions		CT		*T*_1_		*T*_2_		Contrast enhancement	
Single	11 (40%)	Hypodense	14 (48%)	Hypointense	10 (34%)	Hypointense (central)	2 (7%)	Peripheral or ring	10 (34%)
Multiple	18 (60%)	Isodense	3 (10%)	Isointense	5 (17%)	Isointense	2 (7%)	Uniform or patchy	12 (41%)
		Hyperdense	1 (3%)	Hyperintense	5 (17%)	Hyperintense	13 (45%)	No enhancement	4 (14%)
		Heterogeneous	0	Heterogeneous	2 (7%)	Heterogeneous	2 (7%)	Contrast not used	2 (7%)
		Not stated	11 (38%)	Not stated	7 (24%)	Not stated	10 (34%)	Not stated	1 (3%)

**Table 3.** Summary of imaging findings in 29 reviewed cases

FLAIR		DWI		Gradient echo		Haemorrhage (CT or MRI)		Meningeal enhancement	
Hypointense	2 (7%)	Restricted diffusion*^a^*	4 (14%)	Haemorrhage/blooming	3 (10%)	Haemorrhage noted	9 (31%)	Present	4 (14%)
Hyperintense	6 (21%)							Absent	1 (3%)
Isointense	0								
Not stated	21 (72%)	Not stated	25 (86%)	Not stated	26 (90%)	Not stated	20 (69%)	Not stated	24 (83%)

DWI, diffusion-weighted imaging; FLAIR, fluid-attenuated inversion-recovery.

*^a^*Includes one “central restricted diffusion”.

**Table 4.** Summary of imaging findings in 29 reviewed cases

Oedema		Mass effects		Infarction or necrosis		Hydrocephalus	
Cerebral or perilesional	18 (62%)	Midline shift or herniation	14 (48%)	Present	5 (17%)	Present	2 (7%)
No oedema	0	No mass effects	2 (7%)	Absent	1 (3%)	Absent	3 (10%)
Not stated	11 (38%)	Not stated	13 (45%)	Not stated	23 (80%)	Not stated	24 (83%)

Overall, the most frequently reported imaging findings in amoebic encephalitis cases reviewed were

- CT: hypodense mass lesion(s)
- MRI *T*_1_: hypointense
- MRI *T*_2_: hyperintense
- contrast enhancement
- oedema
- haemorrhage
- mass effect.

## Discussion

Four free-living amoebae species have been reported to cause encephalitis in humans: *Acanthamoeba* spp., *Balamuthia mandrillaris*, *Naegleria fowleri* and *Sappinia diploidea*.^6^ Amoebic encephalitis typically affects only immunocompromised patients,^19,20^ although it can occur in immunocompetent healthy individuals, particularly from *N. fowleri*.^3^ The high proportion (38%) of healthy individuals in reviewed cases almost certainly reflects a tendency to publish rarer cases in the literature rather than the true proportion of immunocompetent patients affected.^19^ Amoebic encephalitis has a mortality rate of over 90% with no established effective treatments, although there may be some potential options for certain organisms,^19–22^ and is often a post-mortem pathological diagnosis.^11,20^ Imaging diagnosis is difficult, but our review of the findings in published case reports revealed some “typical” features such as hypointensity on *T*_1 _weighted MRI, hyperintensity on *T*_2 _weighted MRI, contrast enhancement, haemorrhage, oedema and mass effects, but these are not definitive and were not observed in all cases. Furthermore, differential diagnoses such as encephalitis from a different pathogen (viral, bacterial, etc.) tumour (primary or secondary) and infarction also exhibit many of these imaging features. Sound knowledge of the spectrum of imaging findings in amoebic encephalitis is essential for facilitating early accurate diagnosis and providing the patient with the best chance of survival.

From the limited understanding of this rare diagnosis, our case report represents a fairly typical history and series of imaging findings in amoebic encephalitis, including hypointensity on *T*_1_ and hyperintensity on *T*_2_. Contrast enhancement was reported in 75% of cases and was the most consistent finding; however, there were four cases (14%) where contrast was used but the amoebic lesion(s) did not enhance. Haemorrhage on CT, gradient echo or other MRI sequences has also proven to be a particularly common feature, with several authors, including LaFleur et al^8^ (2013), commenting on central low signal on *T*_2_ images to suspect central haemorrhage. Too few cases have described the appearance of haemorrhage on FLAIR, diffusion-weighted imaging, meningeal enhancement, hydrocephalus, infarction or necrosis to estimate its frequency in amoebic encephalitis, but there may be valuable additional diagnostic findings such as hyperintensity on FLAIR, restricted diffusion and meningeal enhancement.

The case series by Singh et al^16^ suggests that there may be variation in imaging findings, depending on the causative organism. The five cases presented had different causative organisms and significant points of difference in their radiological appearance, including density on CT scan and intensity on MRI, but all demonstrated contrast enhancement. Another case series by Galarza et al^4^ reported four paediatric cases of amoebic encephalitis, all with *B. mandrillaris* as the causative organism, with more consistent imaging findings. All four cases were described as hypodense on CT scan and hyperintense on *T*_2_ MRI, with some variation in *T*_1_ intensity and contrast uptake.

## Conclusions

This case demonstrates the clinical presentation, diagnostic uncertainty and fatal outcome typical for the rare disease ameobic encephalitis. The imaging findings in this case and throughout the literature demonstrated that there are several common cerebral imaging findings, but they are inconsistent and may vary depending on the causative organism. From a diagnostic imaging perspective, it is important to have a high level of clinical suspicion of amoebic encephalitis when faced with intracranial mass lesions in an immunocompromised patient.

## Learning Points

1. Amoebic encephalitis is a rare, usually fatal infective disease that generally affects immunocompromised patients.
2. Common findings on cerebral imaging are hypodense mass lesion(s) on CT scan, hypointensity on *T*_1_ MRI, hyperintensity on *T*_2_ MRI, contrast enhancement (may be ring or peripheral), haemorrhage, oedema and mass effects. Additional findings may include hyperintensity on FLAIR images, restricted diffusion and meningeal enhancement.
3. The “typical” imaging findings are not observed in all cases of amoebic encephalitis and there may be variation depending on the causative organism.
4. Diagnostic radiologists must have a high level of suspicion of amoebic encephalitis when faced with intracranial mass lesions in a patient with immunosuppression.

## Consent

The patient presented in the case report is deceased, but the next of kin has provided consent for publication of the case report and images.
